# Expression and Distribution of the Auxin Response Factors in *Sorghum bicolor* During Development and Temperature Stress

**DOI:** 10.3390/ijms20194816

**Published:** 2019-09-27

**Authors:** Dan Chen, Weian Wang, Yaqin Wu, Hui Xie, Linfei Zhao, Qi Zeng, Yonghua Zhan

**Affiliations:** School of Life Science and Technology, Xidian University, Xi’an 710126, China; dchen@xidian.edu.cn (D.C.); weian02.wang@gmail.com (W.W.); wuyq@ascend-bio.com (Y.W.); hxie@xidian.edu.cn (H.X.); linfeizhao1@gmail.com (L.Z.)

**Keywords:** ARF, *Sorghum bicolor*, expression patterns, development, high and low-temperature stresses

## Abstract

Auxin response factor (ARF) is a transcription factor that can specifically bind to the promoter of auxin-responsive genes in plants and plays an important regulatory role in plant growth and development. The previous studies have predicted 25 *ARF* genes in *Sorghum bicolor* (*SbARFs*) and indicated that SbARFs play complex roles in salt and drought stresses. In this study, we reclassified and analyzed the structures of ARFs in three plants, including sorghum, rice, and *Arabidopsis*. Phylogenetic analyses categorized 73 ARF into five classes. By studying the characterization of the structures, it was found that SbARFs from the same evolutionary branches showed similar motif patterns. Furthermore, the expression patterns of *SbARF* genes during development and temperature stress were investigated in sorghum. Quantitative transcription-quantitative polymerase chain reaction (qRT-PCR) results suggested that they had different expression patterns in vegetative and reproductive organs at various developmental stages. High and low-temperature treatments and qRT-PCR demonstrated some of them changed dramatically along with the increase of treatment time. Additionally, in situ hybridization results displayed that *SbARF* genes were accumulated in vascular tissues under temperature stress. These findings provide evidence that SbARFs may play important roles in sorghum vegetative development, reproductive development, and auxin response to temperature stress.

## 1. Introduction

Auxin is known to be involved in the regulation of growth and development and abiotic stress response. As an important family of proteins in auxin-mediated response, ARF can recognize the auxin-response elements (AuxREs) in the promoters of auxin-responsive genes and then activate or repress their expression [1,2]. Most ARF proteins consist of three parts: An N-terminal B3-type DNA binding domain (DBD) that may recognize the AuxREs, a variable middle region that may play as an activation domain (AD) or repression domain (RD), and two C-terminal domains (CTD: domain III/IV). The CTD is involved in protein-protein interactions by dimerizing with auxin/indole-3-acetic acid (Aux/IAA) family genes or even dimerizing between ARFs [1,3,4].

According to the characteristic of molecular structures, ARFs have been identified from different plant species, and their roles in auxin-related plant growth and development have been studied intensively. The first ARFwas identified from *Arabidopsis* (AtARF1) and, subsequently, 23 ARF members were found in *Arabidopsis* [5]. Since then, based on the genome-wide analysis, *ARF* gene family has been identified in more than 20 species of higher plants, such as 25 *ARFs* from rice [6], 36 *ARFs* from maize [7], 24 *ARFs* from chickpea [8], 20 *ARFs* from Tartary buckwheat [9], 39 *ARFs* from litchi [10], and other species. The gain-of-function and loss-of-function mutants help a thorough understanding of the crucial role of ARFs. By using *Arabidopsis* mutants, many studies have been done about it in recent years. As transcriptional repressors, AtARF1 and AtARF2 are involved in floral initiation and abscission, and rosette leaf senescence [11]. Later research has found that AtARF2 represses the cell division and expansion in many vegetative and floral organs [12]. It may also serve as a molecular link with ABA-mediated regulation of seed germination, and root meristem and drought stress [13,14,15]. AtARF3 has confirmed the roles in self-incompatibility, gynoecium patterning, shoot apical meristem maintenance, organ polarity, and regeneration [7,16,17,18,19].

In addition to the functions of organ polarity determination and patterning, recent studies indicated that AtARF3 may have different roles in early flower development [20]. Further gene function analysis revealed that ARF3 controls cell division in the floral meristem by repressing cytokinin biosynthesis and signaling [21]. AtARF5 is reported to regulate gametophyte and embryo development, root growth and leaf vascular differentiation [22,23,24]. Furthermore, sweet potato ARF5 may participate in carotenoid biosynthesis, salt, and drought tolerance in transgenic *Arabidopsis* [25]. AtARF7 regulates hypocotyl response to blue light and auxin stimulation, while AtARF8 affects hypocotyl elongation, auxin homeostasis and fruit development [26,27,28]. In the monocot plant, the first full-length *ARF* gene was cloned from rice and named *ARF1* [29]. The antisense *OsARF1* transgenic rice shows extremely low growth in vegetative and was sterile [30]. At present, function studies have revealed the effect of *ARF* genes in the process of plant growth and development, but there are still many unknown roles. Thus far, compared with other studies, the biological functions of *ARF* genes in temperature stress response are limited [31,32]. *ARF 10/17* genes are induced in anthers of heat stress-insensitive cotton and suppressed in heat stress-sensitive cotton, suggesting the activation of auxin response to anther indehiscence [31]. Microarray and quantitative PCR analyses find that most *OsARF* genes are suppressed by cold and heat stresses in the leaves of rice seedling [32].

Sorghum belongs to the grass family and is a typical annual C_4_ plant. To be a worldwide foodstuff and cash crop, it is distributed throughout the tropical, subtropical, and temperate regions [33]. Sorghum also has the characteristics of rapid growth, heat tolerance, and drought tolerance. The previous studies have predicted 25 *ARF* genes in *Sorghum bicolor* (*SbARF*) and indicated that *SbARFs* may play complex roles in salt and drought stresses [34]. In this study, more effective software (such as Ensembl Plants, MEGA X, MEME, SMART, and InterPro) allow us to reclassify and structure analysis of the *ARFs* genes in three plants, including sorghum, rice, and *Arabidopsis*. The detailed information about *SbARF genes* was provided on sequence structures, characteristic regions, and motif compositions. And we focused on the expression pattern of these *SbARF* genes during development and temperature stress in sorghum. Additionally, in situ hybridization results indicated the location and distribution of *SbARF* genes under temperature treatments. This study provides a different perspective for future regulation mechanism understanding of the auxin response factors in sorghum development and temperature stress.

## 2. Results

### 2.1. Identification of SbARF Genes

The previous studies have predicted 25 *ARF* genes in *Sorghum bicolor* and divided into four distinct classes. To verify the *SbARF* genes, BLASTP approaches were employed for the mining of all putative *SbARF* members in the *Sorghum bicolor* genome. Finally, a total of 25 potential *SbARF* genes were identified which have the same sequence ID as described before (Table 1). Further analysis of the 25 *SbARF* genes revealed that the ORF length of this family ranged between 1557 (*SbARF16*) and 3432 (*SbARF7/25*) bp, with a mean length of 2389 bp, while the transcript length ranged between 1869 (*SbARF2*) and 3836 (*SbARF7*) bp, with a mean length of 2763 bp. The predicted polypeptide length ranged between 518 (SbARF16) and 1143 (SbARF7/25) amino acids, with a mean length of 795 amino acids. The isoelectric point of the ARF proteins ranged between 5.7 (SbARF8) and 8.7 (SbARF2), with a mean value of 6.8. The predicted molecular mass of the proteins ranged between 56 (SbARF16) and 127 (SbARF7) kDa, with a mean molecular mass of 88 kDa.

### 2.2. Phylogenetic Analysis and Classification of SbARF Genes

**Figure 1:**
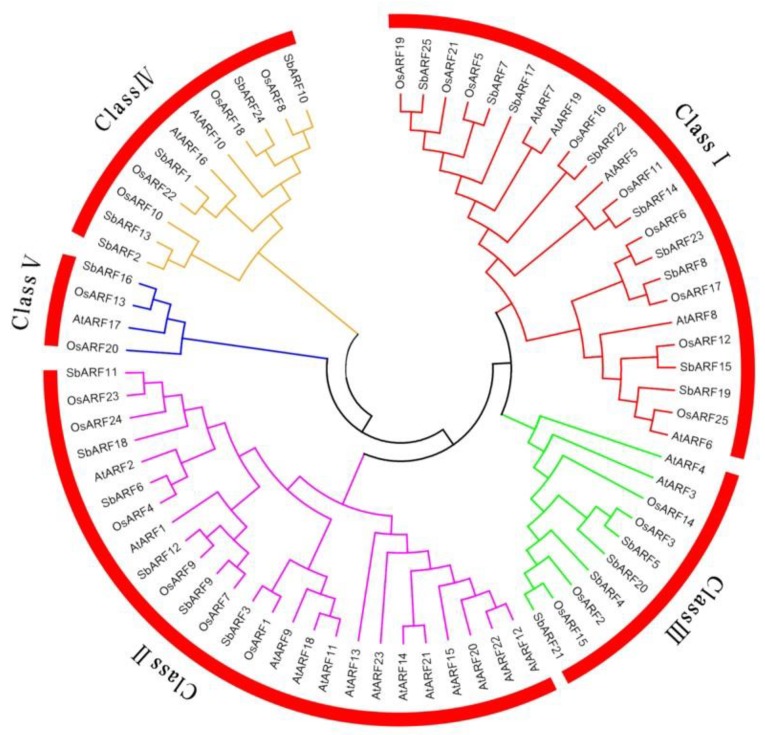
Phylogenetic analysis of ARF in *Sorghum bicolor*, *Oryza sativa* and *Arabidopsis*. The NJ tree shows that the ARFs of *Sorghum bicolor* (25), *Oryza sativa* (25), and *Arabidopsis* (23) were clustered into five classes (I–V). Class I, II, III, IV, and V are marked with red, fuchsia, green, yellow, and blue lines, respectively.

A phylogenetic tree without roots was generated based on multiple alignments of SbARFs with their orthologs from rice and *Arabidopsis* (Figure 1). All 73 ARFs (including 25 SbARFs, 25 OsARFs, 23 AtARFs) fell into five broad groups: Class I, II, III, IV, and V, which contain 23, 25, 10, 11, and four members, respectively. Obviously, most ARFs of the three species were clustered together in Class I and II while Class V contained the fewer members of ARFs. Thus, the 25 SbARFs could also be divided into five separate clusters as well. Class I included SbARF 7/8/14/15/17/19/22/23/25; Class II included SbARF 3/6/9/11/12/18; Class III included SbARF 4/5/20/21; Class IV included SbARF 1/2/10/13/24; Class V only contained one member, SbARF 16. Furthermore, nearly all SbARF were more closely related to rice than *Arabidopsis*.

### 2.3. Gene Structure and Protein Structure of SbARF Genes

**Figure 2:**
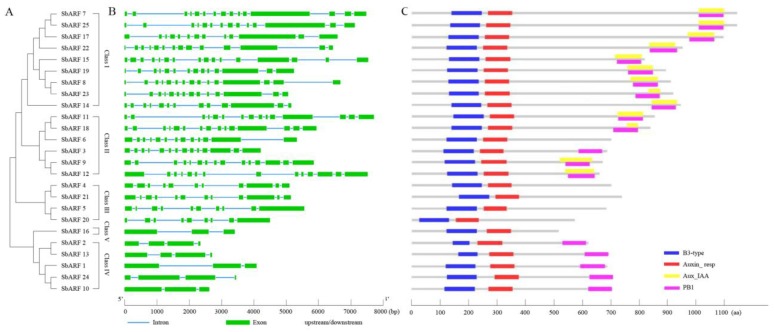
*ARF* gene family in *Sorghum bicolor*. (**A**). Multiple alignments of 25 SbARFs using Clustal W 1.83. The phylogenetic tree was constructed using MEGA X software with a bootstrap analysis of 1000 replicates. (**B**) Exon/intron distribution in the *SbARF* family genes. (**C**) Conserved domain analysis of SbARFs. The length of exons/intron and conserved domain can be estimated using the scale at the bottom, respectively.

To better understanding, the structure of *SbARF* genes, their intron/exon number, positions, and functional domains were analyzed. As shown in Table 1 and Figure 2, the number of exons in DNA sequences varied from 3 to 16, while the number of coding exons varied from 2 to 14. All SbARFs had two characteristic regions: a highly conserved region corresponding to the B3-type DNA-bind domain (DBD) in the N-terminal portion, and a middle region that functions as activation or repression domain (MR). Thirteen of which contained the C-terminal Aux/IAA domains (CTD). Nineteen of which contained the type I/II Phox and Bem1p protein-protein interaction domains (PB1). In general, the *SbARF* genes from the same clusters shared a similar structure. For example, polypeptide length of Class I was the longest, with a mean length of 981 amino acids. Members of Class I and Class II had 13 or 14 coding exons. *ARF* genes from other classes contained fewer exons and encoded proteins of shorter lengths. All proteins from Class I had four characteristic regions (DBD, MR, CTD, PB1), while proteins from Class III, IV, V had only two characteristic regions (DBD, MR).

**Figure 3:**
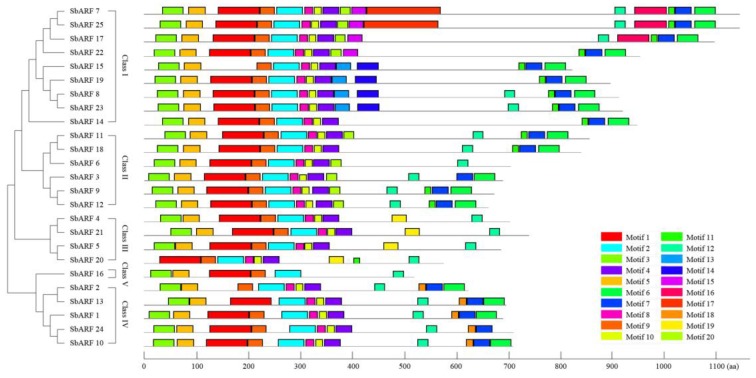
Motif analysis of the SbARF family proteins. Each colored box represents a motif of the protein. The sequence information of 20 motifs is provided in Table S3. The length of proteins can be estimated using the scale at the bottom. The clusters’ information is marked on the left.

A conserved motif analysis of 25 SbARF protein sequences using the MEME online tool predicted 20 motifs (Figure 3). The results displayed, the closer they were in the evolution tree, the more similar location of the motifs would be. Motif 1 was the most conserved motifs with an e-value of 9.6E-1367, and 23 SbARFs had this motif. Twenty-five SbARFs had motif 2, while this motif was also very conservative with the e-value of 7.2 × 10^−^^544^ (Table S3). Besides these, 24 SbARFs contained motifs 3/4/5/8/9/10. Three characteristic domains (DBD, MR, CTD) of SbARFs were divided into 10 motifs. Motif 1 and 9 constituted the DBD. The MR domain consisted of motifs 2, 4, 8, and 10. The CTD corresponded to motifs 6, 7, and 11. Additionally, all the SbARFs were predicted to be nuclear localized using UniProt online analysis tool and subCELlular LOcalization predictor.

### 2.4. Expression of *SbARF* Genes in Different Organs and Tissues

**Figure 4:**
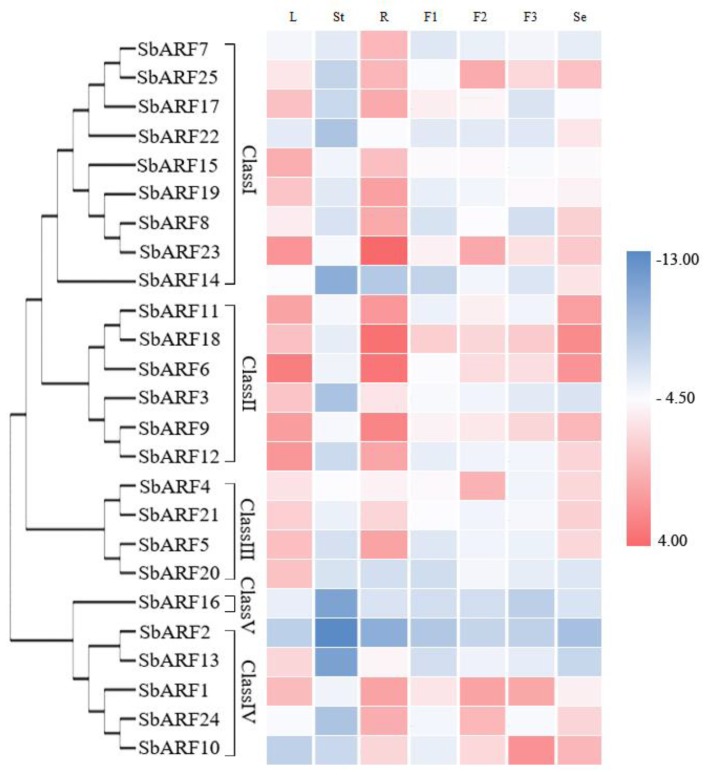
Expression profiles of *SbARF* genes in different tissues. The heatmap was generated based on the relative expression values of 25 *SbARFs* genes obtained by qRT-PCR in seven different tissues and organs. The red and blue color scale represents log2-transformed values and indicates relatively high or low expression, respectively. Every sample is three biological replicates. L, leaves; St, stem; R, root; F1, immature inflorescence; F2, mature inflorescence; F3, post-flowering inflorescence; Se, seeds.

Specific primers were designed to amplify 25 *ARF* genes in *Sorghum bicolor*. The expression levels of *SbARF* genes were analyzed using qRT-PCR in different tissues including vegetative organs (mature leaves, roots, stems) and reproductive organs (immature inflorescence, mature inflorescence, post-flowering inflorescence, and mature seeds) (Figure 4). Generally, most *SbARF* genes showed low expression in mature stems, and high expression in different tissues, except that *SbARF2* and SbARF16 exhibited low transcript levels in all the collected tissues. Some *SbARF* genes including *SbARF3/5/6/7/8/9/12/13/15/17/19/20* showed special higher expressions in the vegetative organs, while *SbARF4/10/14* showed special higher expressions in the reproductive organs. Among these highly expressed genes (15), most of them from Class I (6), Class II (4) and Class III (3). Additionally, transcript accumulation could be detected for several SbARF genes (*SbARF1/11/16/21/22/24/25*) in both vegetative and reproductive organs. For example, *SbARF3/12/13/15/16/F20* genes were expressed more strongly in the leaves than in the other organs. Ten *SbARF* genes (SbARF5/6/7/8/9/17/18/19/23/F24) were strongly induced in the roots. *SbARF14* and *SbARF 22* genes were remarkably enhanced in mature seeds, while *SbARF4/25* and *SbARF10* genes displayed higher expression in mature inflorescence and post-flowering inflorescence, respectively.

### 2.5. Expression of SbARF Genes in Response to Temperature Stress

**Figure 5:**
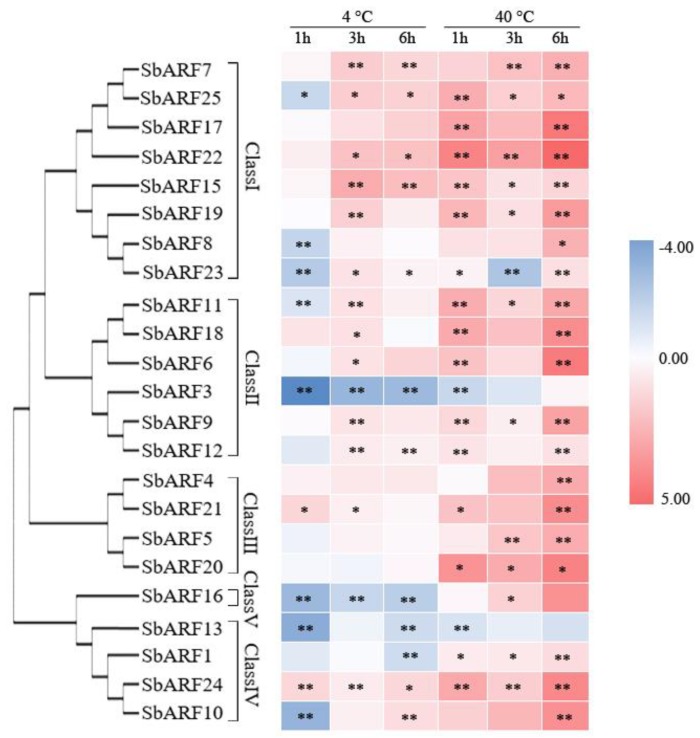
Expression profiles of *SbARF* genes in sorghum seedling under cold and heat stress. The heatmap was generated based on the relative expression values of 25 *SbARFs* genes obtained by qRT-PCR. The expression levels of *ARF* genes under non-stressed conditions were defined as 1. The red and blue color scale represents log2-transformed values and indicates relatively high or low expression, respectively. Data from three biological replicates are displayed with standard deviation. Asterisks on the top of bars indicate significant differences as determined by Student’s *t*-test (*: *p* < 0.05; **: *p* < 0.01).

As transcription factors, ARFs could modulate auxin signaling pathway, and then controlled a wide array of plant behaviors including developmental processes and abiotic stress response. To further determine the role of *SbARF* genes in temperature stress response, expression characteristics of *SbARF* genes were investigated in seedling under 4 °C and 40 °C treatment. As shown in Figure 5 and Figure S1, the expressions of 9 *SbARF* genes (*SbARF4/7/9/15/17/19/21/22/24*) were always up-regulated after cold and heat stress, while *SbARF13* remained down-regulated during the whole treatments. Three *SbARF* genes *(SbARF1/3/16)* showed low expression levels during 4 °C treatment, but 9 *SbARF* genes (*SbARF5/6/8/10/11/12/18/20/25*) displayed different expression patterns. For example, *SbARF11* and *SbARF25* were significantly down-regulated after 1 h of 4 °C treatment and then were rapidly up-regulated after a 3 h treatment. Eleven *SbARF* genes (*SbARF1/5/6/8/10/11/12/16/18/20/25*) showed high expression levels during 40 °C treatment, but *SbARF3* was obviously suppressed after 1 h treatment and then slightly accumulated after 6 h treatment. It was worth mentioning that the expression of the *SbARF23* gene was obvious changed not only at 4 °C but also at 40 °C treatments.

Most of the *SbARF* genes from Class I and Class III displayed transcript accumulation after temperature stress. Instead, the expression levels of *SbARF1/10/13* (Class IV) and *SbARF16* (Class V) were significantly suppressed after treatment. Furthermore, *SbARF13* was the only gene that always maintained a low level.

### 2.6. Distribution Patterns of the *SbARF* Genes under Temperature Stress

**Figure 6:**
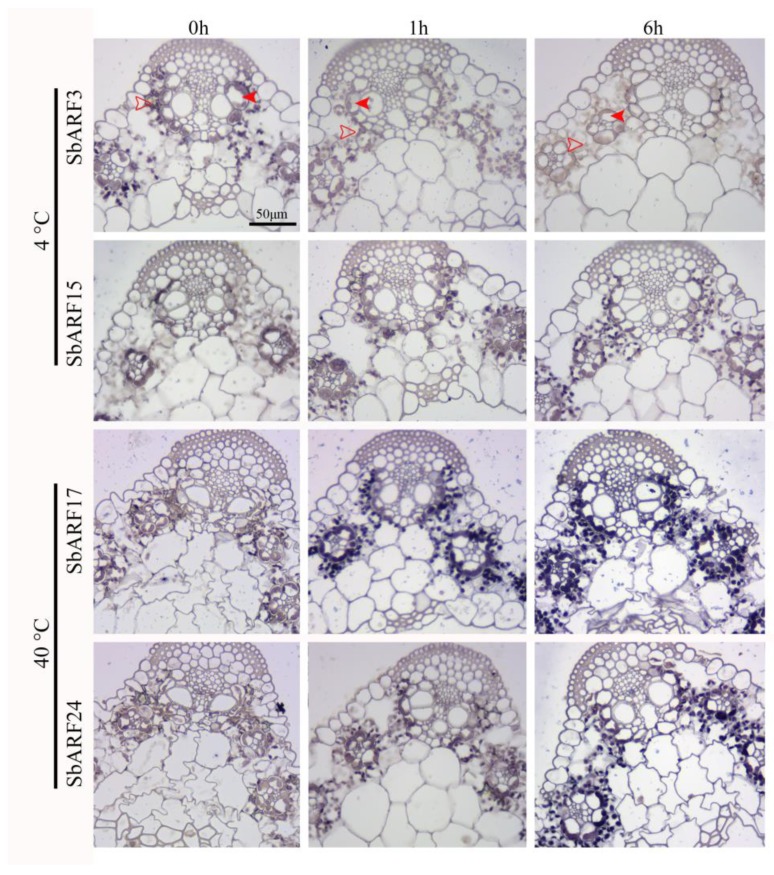
In situ hybridization analysis of *SbARF* genes in seedling leaves under temperature stress. Solid and feint red arrows indicate the regions of vascular bundle sheath cells and the surrounding parenchyma cells, respectively.

To investigate the tissue site of auxin signal response under temperature stress in *Sorghum bicolor*, in situ hybridization analysis was used to detect the distribution patterns of *SbARF* genes in seedling leaves under temperature stress. According to the significant expression change of *SbARFs*, *SbARF3/15/17/24* genes were selected for in situ hybridization analysis. As shown in Figure 6, these *SbARF* mRNAs displayed similar accumulation sites in the leaves. The hybridization signals were accumulated in the vascular bundles of the main and secondary veins, especially in the vascular bundle sheath cells and the surrounding parenchyma cells. The other cells were almost non-detected signals. Although the stain signals were weak after cold stress, they followed a similar distribution pattern no matter heat stress or cold stress.

## 3. Discussion

As a worldwide food and feed cereal crop, sorghum has the characteristics of rapid growth, high-temperature tolerance, and drought tolerance. Here, a total of 25 potential *SbARF* genes were identified, which have the same sequence ID as described before (Table 1). There was the same number of *SbARF* genes as *ARF* genes encoded in *Oryza sativa* (25) [6], but more than *Arabidopsis* (23) [5]. The previous studies have also predicted 25 *ARF* genes in *Sorghum bicolor* [34]. It is worth mentioning that effective bioinformatics methods were used to further analyze the sequence feature in our study, such as Ensembl Plants, MEGA X, MEME, SMART, InterPro, and so on.

We found that 73 ARFs (25 SbARFs, 25 OsARFs, and 23 AtARFs) could be categorized into five classes in phylogenetic analyses (Figure 1). Sorghum is an ideal C_4_ grass model and can be a good complement to C_3_ rice for biofuel crops, animal feed, and forage. In the evolutionary tree of ARFs, there were also showed a more close relationship between monocotyledon *Sorghum bicolor* and *Oryza sativa*. Furthermore, the protein typical domains and conserved motifs analysis showed that SbARFs from the same class might have similar regions (Figure 2 and Figure 3). The typical N-terminal B3-type DNA binding domain (DBD) of ARF protein may recognize and bind to AuxREs in the promoters of auxin response genes [35]. In *Sorghum bicolor*, all 25 SbARFs had this typical DBD domain, and very conservative. As showing in Figure 3, 23 of those DBD domains were constituted by motif 1 and 9. Only ARF2 and ARF13 (members of Class IV) were different because their DBD domains were constituted by motif 9 or motif 2, respectively. Besides the DBD domain, 25 SbARFs also had an Auxin_resp middle region (MR), and most of the MR regions were constituted by motif 2, 4, 8, and 10. Only SbARF16 was different because its MR region was constituted by motif 2. The MR region may act as an activation domain (AD) or repression domain (RD). Although there may be still some confusion on the mechanisms of activation and repression, the ADs are found to be enriched in glutamine (Q), while the RDs are enriched in serine (S), proline (P), and threonine (T) [10,35,36]. Interestingly, motifs 2, 4, 8, and 10 in SbARFs contained amino acids S, T, and P, but not Q. The amino acid composition of MR may contribute to further SbARF functional annotation.

C-terminal domain (CTD) was the third characteristic domain in ARF proteins. It has consisted of a dimerization domain (domain III/IV) and involved in protein-protein interactions by dimerizing with proteins encoded by genes from Aux/IAA family or even dimerizing between ARFs [1,3,4]. However, only 13 of 25 SbARFs (members of Class I and II) contain the typical AUX_IAA domains, while 19 of 25 SbARFs contain a type I/II Phox and Bem1p protein-protein interaction domain (PB1) (Table 1 and Figure 2). Furthermore, The PB1 domains overlapped with the AUX_IAA domains in those 13 SbARFs. Recently researches suggest that PB1 domain can interact with other PB1 via electrostatic interactions, and provides positive and negative interfaces. The underlying protein interaction between ARFs and Aux/IAA also contains a PB1 domain [35,36].

The transcript expression profiles can help us screen for candidate *SbARF* genes with potentially distinct functions. The transcriptional abundance of the *SbARF* genes varied greatly in different vegetative organs and reproductive organs (Figure 2), suggesting that the *SbARF* genes had multiple functions in sorghum growth and development. According to the evolutionary tree analysis, Class II members (*SbARF11/18*, *SbARF9/12*) with the highest homology showed the most similar expression patterns, followed by Class I (*SbARF7/25*, *SbARF8/23*), and other classes had lower similarity, indicating potential functional redundancy in Class I and Class II members. Eleven of 25 *SbARF* genes were accumulated in the roots than other tissues, with five (*SbARF7/8/17/19/23*) in Class I and four (*SbARF6/9/11/18*) in Class II, *SbARF5* in Class III and *SbARF24* in Class IV. Seven of 25 *SbARF genes* were accumulated in the leaves, with *SbARF15* in class I, *SbARF3/12* in Class II, *SbARF20/21* in Class III, *SbARF13* in Class IV, *SbARF16* in Class V. *SbARF4* (Class III) and *SbARF25*(Class I) genes were highly induced in the mature inflorescence. *SbARF10* gene (Class IV) was remarkably increased in the post-flowering inflorescence. *SbARF14* and *SbARF22* genes (both members of Class I) were highly expressed in the seeds than other tissues. SbARFs shared remarkable homology with OsARFs as showed in Figure 1. It has been reported that the transgenic antisense-*OsARF1* rice grew slowly and leaves were short and curly [30], while its sorghum homolog, *SbARF3*, was prominently higher expressed in the leaves, implying its possible function in vegetative development. The knockout mutant of *OsARF12* and double mutant of *OsARF12/25* display unusual growth of seedling, especially in root growth [37,38]. Thus, their sorghum homologs *SbARF15* and *SbARF19* genes may be important for vegetative growth in sorghum, along with obviously higher accumulation in both leaves and roots, but lower in reproductive organs. However, different from its rice homolog *OsARF16* gene, which is required for auxin responses in the roots [39], the expression of the *SbARF22* gene peaked in the mature seeds, and then in the roots. The evidence of the similarities and differences between the transcription levels may imply the evolution and divergence of *Sorghum bicolor* and *Oryza sativa* in some way.

Sorghum is a typical C_4_ grass model crop adapted to heat stress [33]. Auxin is well-known for its numerous functions in plant morphogenesis and plant tolerance to stresses, including heat stress and cold stress [31,32]. It was reported that *ARF10* and *ARF17* genes are induced in anthers of heat stress-insensitive cotton and suppressed in heat stress-sensitive cotton [31]. In rice, microarray and quantitative PCR analyses find that most *OsARF* genes are suppressed by cold and heat stresses, while *OsARF4/14/18/19* and *OsARF11/13/16* are induced by cold and heat stresses, respectively [32]. It’s worth mentioning that their sorghum homologs *SbARF6/24/25* and *SbARF16/22* also were significantly induced by cold and heat stresses, respectively. Furthermore, *SbARF* genes with the highest homology in Class I, Class II, and Class III showed similar accumulation pattern in normal growth and development, except members in Class IV and Class V. In temperature stress, members of Class I and Class III showed similar accumulation pattern, while some members of Class II and Class IV had different expression patterns. The finding also supported the potential functional redundancy in Class I members and no only that, those genes (such as members in Class I/II/IV/V) that the expression levels changed significantly may have special roles in temperature stress. Clearly, most of *SbARF* genes were induced by heat stress, which possibly indicated the participation of auxin in the high temperature resistant of sorghum.

Since ARFs are important proteins in auxin-mediated response, which effect the activation of Aux/IAA and PIN, the in situ hybridization might point out the auxin response site in tissues under temperature stress. We found that no matter heat or cold stress, the distribution patterns of *SbARF* mRNAs were similar in the seedling leaves (Figure 6). They mainly expressed in the vascular bundle sheath cells and the surrounding parenchyma cells. The distribution pattern of vascular tissue accumulation is consistent with the accumulation of PINs and auxin in the vascular strands of *Arabidopsis* [40,41,42,43]. It is reported that in the loss-of-function and gain-of-function *AtARF5* mutation, not only PIN1 expression is changed, but also the vascular tissue formation is abnormal [22,23]. Auxin efflux carrier PIN3/4/6/7/8 can be also detected in the vascular tissues of *Arabidopsis*, a green signal of PIN3-GFP presents primarily in the xylem parenchyma cells, PIN4/7 expressed in the cambium, PIN6 expressed in the cambial cells adjacent to the xylem, and PIN8 expressed in the cambial cells near PIN6 [41]. It is hypothesized that these *SbARF* genes have participated in auxin response to temperature stress in the vascular tissues.

## 4. Materials and Methods

### 4.1. Identification and Phylogenetic Analysis of *SbARF* Genes

The *Sorghum bicolor* genome was downloaded from the Resources for Plant Comparative Genomics (PlantGDB; Available online: http://www.plantgdb.org/). The BLASTP method was adopted to search for the *SbARF* genes and to determine the maximum number of genes. The sequences of 25 *Oryza sativa ARF* and 23 *Arabidopsis ARF* genes were downloaded from the Plant Transcription Factor Database (PlantTFDB; Available online: http://planttfdb.cbi.pku.edu.cn/) [5,6]. These ARFs were used as bait to identify SbARF, via a BLASTP search at the score value of ≥100 and e-value ≤1 × 10^−^^5^. Then all identified potential ARFs were further validated and analyzed by Ensembl Plants (available online: http://plants.ensembl.org/) and PFAM database (available online: https:// www.pfam.xfam.org/). Finally, 25 *SbARFs* genes were identified for further analysis. The protein sequences were investigated by multiple alignment analyses using Clustal W. The resultant sequence alignment then served as input data for a neighbor-joining phylogenetic tree constructed using the MEGA X software [44] with a bootstrap analysis of 1000 replicates.

### 4.2. Gene Structure and Protein Structure Analysis

The mRNA sequences of *SbARF* genes were obtained from PlantTFDB. The DNA sequence information and protein information were predicted online using Ensembl Plants and SMART (available online: http://smart.embl-heidelberg.de/). The conserved motifs were predicted by the MEME website (available online: http://alternate.meme-suite.org/tools/meme) [45] with the following parameters: optimum motif width was 6 to 200, and the maximum number of motifs was 20. The InterPro online program (available online: https://www.ebi.ac.uk/interpro/) was used to further analyze and annotate the protein sequences. To predict the function and subcellular location of SbARFs, the UniProt online analysis tool (available online: https://www.uniprot.org/) and CELLO v.2.5: subCELlular LOcalization predictor (available online: http://cello.life.nctu.edu.tw/) were used.

### 4.3. Plant Materials and Treatment Methods

The tissues and organs from the adult *Sorghum bicolor* used for gene expression analysis were collected in the experiment field at Xidian University (Xi’an, China). Leaves (L), roots (R), stems (St), immature inflorescence (one week before flowering, F1), mature inflorescence (flowers at full bloom, F2), post-flowering inflorescence (one week after flowering, F3), and mature seeds (Se) were collected from seven-week-old plants. The samples were performed according to the previous description [46]. For the temperature stress treatments, sorghum seeds were grown in Murashige and Skoog medium in an illumination incubator at 24 ± 2 °C with 16-h light/8-h dark cycles. Fourteen-day-old seedlings were treated with 40 °C as heat stress or 4 °C as cold stress for 1 h, 3 h, and 6 h, and then were collected immediately. Each experiment was repeated three times and all the materials were stored at −80 °C until RNA extraction.

### 4.4. Quantitative RT-PCR Analysis

Total RNA was extracted using RNAiso Plus (TaKaRa, Tokyo, Japan) according to the manufacturer’s instructions, cDNA was synthesized using the Reverse Transcription System (TaKaRa, Tokyo, Japan). Quantitative RT-PCR (qRT-PCR) analysis was performed in a 7300 Real-Time System (ABI, NewYork, American) using the SYBR Green RealMasterMix (TIANGEN, Beijing, China). The qRT-PCR primers were designed by Primer 5 software (Table S1). The housekeeping gene, *Actinβ*, was used as internal control. The quantification of each sample of cDNA was performed in triplicate, each PCR was replicated three times for verification, and the 2^−ΔΔCT^ method was used to analyze the relative changes in gene expression from the qPCR experiments.

### 4.5. Paraffin Section of Leaves and in Situ Hybridization

For fine localization of *SbARF* mRNA signals in leaves under temperature stress, paraffin section and in situ hybridization analysis were used after 4 °C and 40 °C treatments. The 14-day old seedlings of *Sorghum bicolor* were obtained after 1 h and 6 h temperature stress treatments. The leaf tissues were collected and fixed with FAA (70% alcohol: glacial acetic acid: formalin = 18:1:1, made with diethylpyrocarbonate-treated water) for 1 h. Then the samples were dehydrated and embedded paraffin. The leaf sections were cut at a 10 mm thickness under a rotary microtome (Leica, Wetzlar, Germany) mounted on poly-Lys-coated glass slides. The gene-specific probe was amplified with the primers Probe-SbARF-F and Probe-SbARF-R (Table S2), and the mouse anti-digoxigenin-labeled alkaline phosphatase (anti-DIG-AP) was added dropwise. Pretreatment, hybridization and immunological detection of sections were performed as described [47].

## 5. Conclusions

The systematic characterization of the *ARF* gene family in sorghum has revealed key features about their structures and the potential functions of the *SbARF* gene family in vegetative and reproductive organs. High and low-temperature treatments and qRT-PCR demonstrated some *SbARF* genes changed dramatically along with the increase of treatment time. Additionally, in situ hybridization results indicated the location and distribution of *SbARF* genes under temperature stress. Our findings provide evidence for future understanding of ARFs in sorghum development and temperature stress.

## Figures and Tables

**Table 1:** *SbARF* gene family information.

*SbARF* gene	Sequence ID	Chr.Location	Strand Direction	ORF Length (bp)	Transcript Length (bp)	No. Exons	No. Coding Exons	UniProt	Protein Length (aa)	MW (Da)	PI	Domains
*SbARF1*	Sb01g019130.1	Chr1:20265083–20269209	Reverse	2070	2354	3	3	C5WYD5	689	75,404.92	6.9	DBD, MR, PB1
*SbARF2*	Sb02g032210.1	Chr2:66973093–66975465	Reverse	1869	1869	4	4	C5X7P6	622	68,290.21	8.7	DBD, MR, PB1
*SbARF3*	Sb03g000530.1	Chr3:375293–379543	Reverse	2067	2712	14	14	C5XJJ7	688	76,895.05	6.4	DBD, MR, PB1
*SbARF4*	Sb03g030740.1	Chr3:58980057–58985203	Reverse	2109	2418	11	10	C5XG18	702	77,003.66	6.4	DBD, MR
*SbARF5*	Sb03g034850.1	Chr3:62997617–63003214	Reverse	2058	2584	10	10	─	685	74,377.98	7.2	DBD, MR
*SbARF6*	Sb03g044630.1	Chr3:71975563–71980937	Forward	2115	2729	13	13	C5XH00	704	78,702.14	7.4	DBD, MR
*SbARF7*	Sb04g003240.1	Chr4:3072716–3080254	Reverse	3432	3836	15	14	C5XUJ9	1143	127,041.26	6.3	DBD, MR, CTD, PB1
*SbARF8*	Sb04g004430.1	Chr4:4219215–4225954	Forward	2736	3126	15	14	C5XVH8	911	100,417.56	5.7	DBD, MR, CTD, PB1
*SbARF9*	Sb04g022830.1	Chr4:52419119–52425028	Reverse	2019	2809	15	14	C5XUU5	672	74,930.18	6.2	DBD, MR, CTD, PB1
*SbARF10*	Sb04g026610.1	Chr4:56508838..56511492	Forward	2127	2487	3	3	C5XXU7	708	76,087.88	7.1	DBD, MR, PB1
*SbARF11*	Sb05g019540.1	Chr5:47874380–47882162	Reverse	2568	3071	15	14	─	855	93,782.34	6.5	DBD, MR, CTD, PB1
*SbARF12*	Sb06g017490.1	Chr6:46853929–46861508	Forward	1986	3151	14	14	C5Y8U9	661	73,201.76	6.1	DBD, MR, CTD, PB1
*SbARF13*	Sb06g022810.1	Chr6:52026442–52029170	Forward	2088	2088	4	4	C5YCE3	695	75,065.79	8.2	DBD, MR, PB1
*SbARF14*	Sb06g031900.1	Chr6:60209330–60214539	Reverse	2841	2841	13	13	C5Y9Z0	946	103,749.51	6.2	DBD, MR, CTD, PB1
*SbARF15*	Sb06g032500.1	Chr6:60654734–60662333	Forward	2466	3084	16	14	C5YA53	821	90,757.87	6.5	DBD, MR, CTD, PB1
*SbARF16*	Sb06g033970.1	Chr6:61857871–61861319	Reverse	1557	1928	3	2	C5YB36	518	56,284.76	6	DBD, MR
*SbARF17*	Sb07g027080.1	Chr7:62218509–62225164	Reverse	3288	3716	13	13	C5YIB6	1095	121,248.57	6.5	DBD, MR, CTD, PB1
*SbARF18*	Sb08g014320.1	Chr8:37871039–37877027	Reverse	2520	3050	15	14	C5YNM8	839	92,454.03	6.7	DBD, MR, CTD, PB1
*SbARF19*	Sb08g021460.1	Chr8:53122868–53128155	Reverse	2688	3063	14	14	C5YRZ9	895	98,609.5	5.9	DBD, MR, CTD, PB1
*SbARF20*	Sb09g025500.1	Chr9:54931364–54935901	Forward	1728	2002	9	8	C5Z0X8	575	63,169.94	7.5	DBD, MR
*SbARF21*	Sb09g028450.1	Chr9:57360258–57365448	Forward	2220	2483	12	11	C5YVJ4	739	80,854.09	7.7	DBD, MR
*SbARF22*	Sb10g006440.1	Chr10:5930430–5936935	Forward	2859	2859	14	14	C5Z600	952	105,068.55	6.4	DBD, MR, CTD, PB1
*SbARF23*	Sb10g027220.1	Chr10:56924176–56929286	Reverse	2760	2760	14	14	C5Z7U5	919	101,692.05	6.4	DBD, MR, CTD, PB1
*SbARF24*	Sb10g027790.1	Chr10:57623853–57627338	Forward	2130	2493	4	3	C5Z8A5	709	76,700.16	7.7	DBD, MR, PB1
*SbARF25*	Sb10g029130.1	Chr10:58946685–58953873	Forward	3432	3574	14	14	C5Z981	1143	126,297.21	6.6	DBD, MR, CTD, PB1

## Supplementary Materials

Supplementary materials can be found at https://www.mdpi.com/1422-0067/20/19/4816/s1.

## Abbreviations

ARF	Auxin response factor
qRT-PCR	Reverse transcription-quantitative PCR
AuxREs	Auxin-response elements
DBD	N-terminal B3-type DNA binding domain
AD	Activation domain
RD	Repression domain
CTD	C-terminal domains
Aux/IAA	Auxin/indole-3-acetic acid
MW	Molecular weight
PI	Isoelectric point
