# Supplementary material for: Expression and Distribution of the Auxin Response Factors in Sorghum bicolor During Development and Temperature Stress
Source: Int J Mol Sci. 2019 Sep 27;20(19):4816. doi: 10.3390/ijms20194816 (PMC6801764; doi:10.3390/ijms20194816)
Supplement: Supplementary file 1 [file ijms-20-04816-s001.pdf]

Supplementary

Table 1. Details of primers used in the qRT-PCR analysis in the study.

Gene	Primers
β-actin	5'ACGGCCTGGATGGCGACGTACATG 3'
	5'GCAGAAGGACGCCTACGTTGGTGAC 3'
SbARF1	5'TTCCGAGAGCCCGACGAGTT 3'
	5'TGCGGCCTCAACAACATCC 3'
SbARF2	5'CGCCGTCAAGGAGTCCATG 3'
	5'GCCCGTCCGAAGAGCATTAT 3'
SbARF3	5'CGCGTTTGTCTACCTGAGGAGTG 3'
	5'CGGGTATGTTGATGGCAGGTG 3'
SbARF4	5'CGCCGTGTGTCTGGAGCTG 3'
	5'GGCCGTGAGCGTCTTGCAG 3'
SbARF5	5'GGCGACGGACGAGGTGTAC 3'
	5'CCAAGCCTTAGTTCACCATCATC 3'
SbARF6	5'GGACGAGCCCTTCAGAGTTCAT 3'
	5'CGTTGCTTTCAGTCAGGTTGTTC 3'
SbARF7	5'TGGGCAGCCTCGTCGTCTAC 3'
	5'CGCCCACGAAAAGACTCCAC 3'
SbARF8	5'TTGCCTGCGGTCGGTAGTC 3'
	5'TCGGCGTGGAACAGAGAATC 3'
SbARF9	5'TGGGCCGTTGGTCACAGTG 3'
	5'TGCCCTCGAAAGATGTGACG 3'
SbARF10	5'GGCGGACGAGCAGGAGAAG 3'
	5'GCGGAGGAACACGATGGAGTC 3'
SbARF11	5'GCTGCTCCGAAGGTCACATTAG 3'
	5'TTGCCTTGCTGGTCTTGATAATG 3'
SbARF12	5'GGCGAGCGCGTCTATTACTTC 3'
	5'CGCCGTGAGTGCTCGTATCT 3'
SbARF13	5'ACGCCGCCCTGGTGAAGAC 3'
	5'CGGGCTGGAGCTTCTTGTTG 3'
SbARF14	5'GCGGCAATGTCTTCCTAGTTATG 3'
	5'CTCCCGTGCAGAAATTGAATC 3'
SbARF15	5' CTGCCAGCTCCATGATGTCAC 3'
	5' CCTCGGAAGATATGCCTGAATT 3'

SbARF16	5' CGCGGACGACAAGACCAAC 3'	
	5' GGCGCATGAAGACGACGAT 3'	
SbARF17	5' GCCCATGCAGCAGCTAATAACAG 3'	
	5' TGGCGAGGGATAAATGAAGAATG 3'	
SbARF18	5' TGCGCCACCAACAAATGAGG 3'	
	5' GCCGAACCCCAACACGTAAC 3'	
SbARF19	5' GCCGGGTCGTCTACTTTCCTC 3'	
	5' CGGCGCGGAACAGAGAATC 3'	
SbARF20	5' GGCGGAGGATAAGATGTTTGG 3'	
	5' GCTCGCCATCACTACCTCTCAG 3'	
SbARF21	5' CCGCAGGCACCTTTTAACC 3'	
	5' TTGGCTTAATCTGGGGTTGTAGT 3'	
SbARF22	5' CGCCGTGCAGCTGAGAAGATAT 3'	
	5' GCCTGTGATCGTGCCCATGTAT 3'	
SbARF23	5' CGCAAGAACTGAAGGACCCATAT 3'	
	5' GCCCTTGGGTTGTAGAAAATTGT 3'	
SbARF24	5' CGCCGGGTTCTCCATGTTC 3'	
	5' GCGGAGACAGTGCCCATGAAC 3'	
SbARF25	5' CCGCCACATATATCGAGGTCAG 3'	
	5' CGCCATTGTGAGTTTTTCCATCT 3'	

Table 2. The gene-specific probes used in the situ hybridization.

Gene	Probes
ARF3	TCCCCTACTCCCACAGTCATCTTGTCAGCAATGCCGCTACTCCTA
ARF15	GCAGTGGGAACAGGGATGGATACATAGGAAAGGTTGTCAGTGGCT
ARF17	CGAAAGAATCCTGATGCACCGGACGCAGT
ARF24	GCACGGTCTGGACGGGCGGGTCGGCGGAGTAG

Table 3. The sequence information of 20 motifs.

Discovered Motifs	Sequences	E-value	Sites	Width
1	SFCKTLTASDTSTHGGFSVPRRAAEKCFPPLDYSQQPPVQELVAKDLHGNEWKFRHIYRGQPRRH LLTTGWSVFVSAKRL	9.6×10^{-1367}	23	80
2	SVLSSDSMHJGVLAAAAHAAATGSPFTVFYNPRASPSEFVVPLAKYLKALY	2.7×10^{-535}	25	51
3	CLNSELWHACAGPLVSLPPVGSLVYYFPQGHSEQVAASTBK	7.0×10^{-522}	24	41

4	WPNSKWRSLKVGWDEPTAGZRPNRVSPWEIEP	6.0×10^{-404}	24	32
5	PNLPPKLJCRVHNVTLHADPDTDEVYAQITLQP	1.5×10^{-390}	24	33
6	WKLVYVDREGDVLLVGDDPWEEFVNSVRCIKILSPEEVQQM	8.8×10^{-339}	18	41
7	VGRSLDJTRFSGYEELRAELARMFGIEGZLED	3.60×10^{-219}	19	32
8	ISVGMRFKMRFETEESS	2.00×10^{-201}	24	17
9	VAGDSVLFJRGEBGZLRLGIRRANRQQTN	3.90×10^{-306}	24	29
10	RRFMGTITGISDLDP	7.70×10^{-122}	24	15
11	RVRTFTKVYKSGS	2.10×10^{-45}	14	13
12	KDQEVKPSSCKLFGISIDSEE	2.10×10^{-37}	21	21
13	LTTFPMYPSPFPLRLKRPWPSGLPSLHGG	6.90×10^{-27}	4	29
14	GNPGFQSLNFQGLGISPWMQPRLDPSLLGLQPDMYQAMAAAA	3.60×10^{-26}	4	42
15	DSSEMENLFKRAMPWLGEEICIKDVQTQN	5.60×10^{-24}	4	29
16	KYQBHJSADIDGNYRIPKDAQQEISSSMVSQSFGVSDIAFNSIDSAINDGPFLNRNSWPPAP	1.30×10^{-28}	3	62
17	TVMPGLSLVQWMNMNRQQNSSFGNSGIQSEYLRSLSNPNMQNLGAAELARQLNMQNQJLQQ NSIQFNAPKLPQQMQPINELAKGSLPCNQLGVGTKQQEQ	1.90×10^{-18}	2	143
18	GLEPGQCKVFVESET	3.50×10^{-18}	5	15
19	GNPGFSYQCSGFGESVRFQEVLQGQEVFR	8.20×10^{-17}	4	29
20	AAPFFIPPPPFFRPKRPRQPA	1.00×10^{-15}	9	21

Background Source : A Alanine, C Cysteine, D Aspartic acid, EGlutamic acid, F Phenylalanine, G Glycine, H Histidine, I Isoleucine, K Lysine, L Leucine, M Methionine, N Asparagine, P Proline, Q Glutamine, R Arginine, S Serine, T Threonine, V Valine, W Tryptophan, Y Tyrosine.

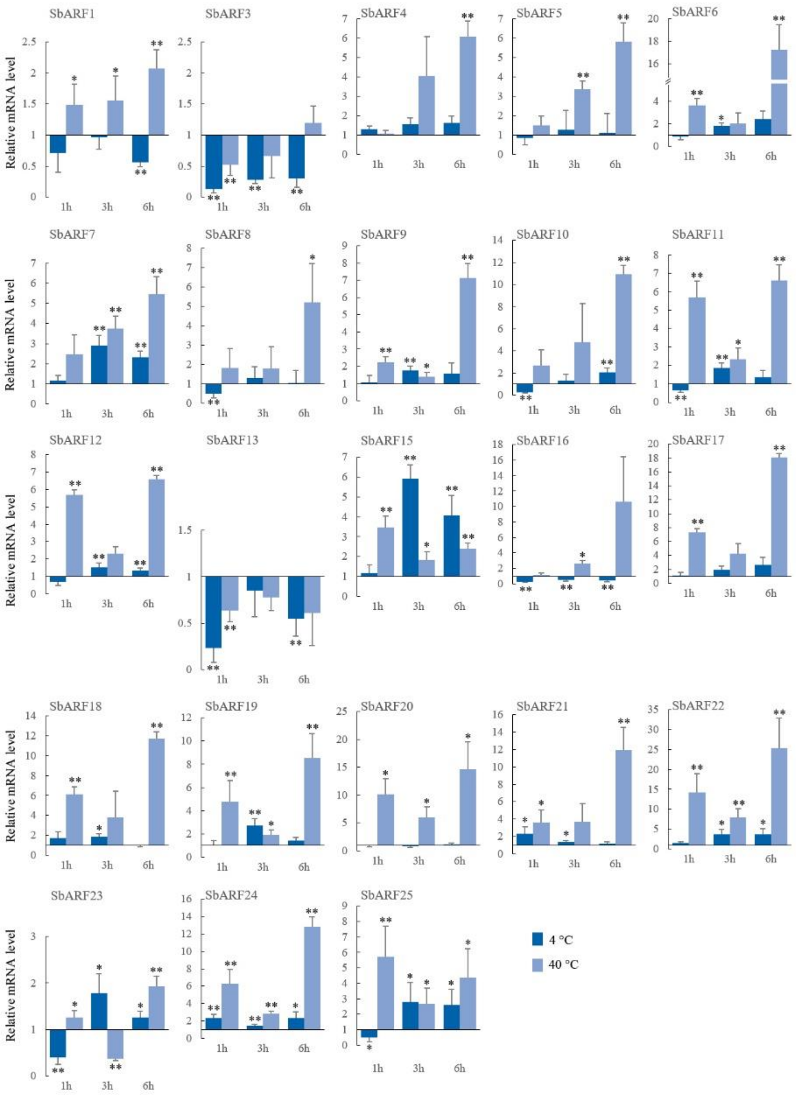

Figure S1. Expression of *SbARF* genes in sorghum seedling under cold and heat stress. Data from three biological replicates are displayed with standard deviation. The expression level of *ARF* gene under non-stressed condition was defined as 1. Asterisks on the top of bars indicate significant differences as determined by Student's t-test (*: $p < 0.05$; **: $p<0.01$).
